# Bochner integrals in ordered vector spaces

**DOI:** 10.1007/s11117-016-0454-9

**Published:** 2016-12-01

**Authors:** A. C. M. van Rooij, W. B. van Zuijlen

**Affiliations:** 1 Department of Mathematics, Radboud University Nijmegen, P.O. Box 9010, 6500 GL Nijmegen, The Netherlands; 2 Mathematical Institute, Leiden University, P.O. Box 9512, 2300 RA Leiden, The Netherlands

**Keywords:** Bochner integral, Ordered vector space, Ordered Banach space, Closed cone, Generating cone, 28B05, 28B15

## Abstract

We present a natural way to cover an Archimedean directed ordered vector space *E* by Banach spaces and extend the notion of Bochner integrability to functions with values in *E*. The resulting set of integrable functions is an Archimedean directed ordered vector space and the integral is an order preserving map.

## Introduction

We extend the notion of Bochner integrability to functions with values in a vector space *E* that may not itself be a Banach space but is the union of a collection $\mathbb {B}$ of Banach spaces.

The idea is the following. We call a function *f*, defined on a measure space *X* and with values in *E*, “integrable” if for some *D* in $\mathbb {B}$ all values of *f* lie in *D* and *f* is Bochner integrable as a function $X \rightarrow D$. Of course, one wants a certain consistency: the “integral” of such an *f* should be independent of the choice of *D*.

In [18], Thomas obtains this consistency by assuming a Hausdorff locally convex topology on *E*, entailing many continuous linear functions $E \rightarrow \mathbb {R}$. Their restrictions to the Banach spaces that constitute $\mathbb {B}$ enable one to apply Pettis integration, which leads to the desired uniqueness.

Our approach is different, following a direct-limit-like construction. We assume *E* to be an ordered vector space with some simple regularity properties (Archimedean, directed) and show that *E* is the union of a certain increasing system $\mathbb {B}$ of Banach spaces with closed, generating positive cones (under the ordering of *E*). Uniqueness of the integral follows from properties of such ordered Banach spaces. Moreover, the integrable functions form a vector space and the integral is linear and order preserving.

In Sect. 3 we study ordered Banach spaces with closed generating cones. We give certain properties which can be used to give an alternative proof of a classical theorem which states that every order preserving linear map is continuous, and generalise it to order bounded linear maps. In Sect. 4 we study Bochner integrable functions with values in an ordered Banach space with closed generating cone. In Sect. 5 we present the definition of a Banach cover and the definition of the extension of the Bochner integral to functions with values in a vector space that admits a Banach cover. In Sect. 6 we show that an Archimedean ordered vector space possesses a Banach cover consisting of ordered Banach spaces with closed generating cones. In Sect. 7 we study integrable functions with values in Archimedean ordered vector spaces. In Sect. 8 we compare the integral with integrals considered in [16]. In Sect. 9 we present an application to view the convolution as an integral.

## Notation

$\mathbb {N}$ is $\{1,2,\ldots \}$. We write “for all *n*” instead of “for all $n\in \mathbb {N}$”. To avoid confusion:

- An “order” is a “partial order”.
- We call an ordered vector space *Archimedean* (see Peressini [15]) if for all $a,b\in E$ the following holds: if $na \le b$ for all $n\in \mathbb {N}$, then $a\le 0$. (In some places, e.g., Birkhoff [4], such spaces are said to be ‘integrally closed’.)

As is common in literature, our notations do not distinguish between a function on a measure space and the class of that function.

## Ordered Banach spaces with closed generating cones

In this section we describe properties of ordered Banach spaces with closed generating cones. Using these properties we prove in Theorem 3.11 that an order bounded map between ordered Banach spaces with closed generating cones is continuous.

### Definition 3.1

An *ordered locally convex vector space* is a locally convex vector space with an order that makes it an ordered vector space. A *normed ordered vector space* is a normed vector space with an order that makes it an ordered vector space. An *ordered Banach space* is a Banach space that is a normed ordered vector space.

A priori there is no connection between the ordering and the topology of an ordered locally convex vector space. One reasonable and useful connection is the assumption that the (positive) cone be closed.

### Theorem 3.2

Let *E* be an ordered locally convex vector space. $E^+$ is closed if and only if

1 $$\begin{aligned} x \le y \iff \alpha (x) \le \alpha (y) \text{ for } \text{ all } \alpha \in (E')^+. \end{aligned}$$

Consequently, whenever $E^+$ is closed then $(E')^+$ separates the points of *E* and *E* is Archimedean.

### Proof

Since $E^+$ is convex, $E^+$ is closed if and only if it is weakly closed (i.e., $\sigma (E,E')$-closed); see [7, Theorem V.1.4]. The rest follows by [1, Theorem 2.13 (3 & 4)]. $\square$

The following theorem is due to Andô [2]. See also [1, Corollary 2.12].

### Theorem 3.3

Let *D* be an ordered Banach space with a closed generating^1^ cone $D^+$. There exists a $C>0$ such that

2 $$\begin{aligned} C\Vert x\Vert&\ge \inf \{ \Vert a\Vert : a\in D^+, - a \le x \le a\} \quad (x\in E). \end{aligned}$$

### Definition 3.4

Let *D* be a directed^2^ ordered Banach space. If $C>0$ is such that (2) holds, then we say that the norm $\Vert \cdot \Vert$ is *C-absolutely dominating*.^3^ We say that a norm $\Vert \cdot \Vert$ is *absolutely dominating* if it is *C*-absolutely dominating for some $C>0$.

On a Banach lattice the norm is 1-absolutely dominating. Actually for Banach lattices there is equality in (2).

### 3.5

We refer the reader to Appendix 1 for the following facts: If $\Vert \cdot \Vert$ is *C*-absolutely dominating on a directed ordered Banach space *D*, then $C\ge 1$. Whenever there exists a absolutely dominating norm, then for all $\varepsilon >0$ there exists an equivalent $(1+\varepsilon )$-absolutely dominating norm. All norms on a directed ordered vector space *D* that make *D* complete and $D^+$ closed are equivalent (see 6.2).

### 3.6

Let *D* be a directed ordered Banach space. Then $\Vert \cdot \Vert$ is absolutely dominating if and only if the (convex) set

3 $$\begin{aligned} \bigcup _{a\in D^+, \Vert a\Vert \le 1} [-a,a] \end{aligned}$$

is a neighbourhood of 0.

### Definition 3.7

(See [12, §16]) Let *E* be an ordered vector space. We say that a sequence $(x_n)_{n\in \mathbb {N}}$ in *E* *converges uniformly* to an element $x\in E$ (notation: $x_n \xrightarrow {u}x$) whenever there exist $a\in E^+$, $\varepsilon _n \in (0,\infty )$ with $\varepsilon _n\rightarrow 0$ and

4 $$\begin{aligned} -\varepsilon _n a \le x_n -x \le \varepsilon _n a \quad (n\in \mathbb {N}). \end{aligned}$$

Note that one may replace “$\varepsilon _n \rightarrow 0$” by “$\varepsilon _n \downarrow 0$”. If *E* is Archimedean then the *x* as above is unique. We will only consider such convergence in Archimedean spaces. We say that a sequence $(x_n)_{n\in \mathbb {N}}$ in *E* is a *uniformly Cauchy sequence* if there exists an $a\in E^+$ such that for all $\varepsilon >0$ there exists an *N* such that $-\varepsilon a \le x_n - x_m \le \varepsilon a$ for all $n,m\ge N$. *E* is called *uniformly complete* whenever it is Archimedean and all uniformly Cauchy sequences converge uniformly.

### Lemma 3.8

Let $D_1,D_2$ be ordered vector spaces and $T: D_1 \rightarrow D_2$. If *T* is linear and order bounded, then *T* preserves uniform convergence.

### Proof

Suppose $x_n\in D_1, \varepsilon _n \in (0,1)$ and $a\in D_1^+$ are such that $-\varepsilon _n a \le x_n \le \varepsilon _n a$ and $\varepsilon _n \downarrow 0$. Let $b\in D_2^+$ be such that $T[- a , a] \subset [-b,b]$. Then $\varepsilon _n^{-1}Tx_n \in [-b,b]$, i.e., $-\varepsilon _n b \le Tx_n \le \varepsilon _n b$ for all *n*. $\square$

### Theorem 3.9

Let *D* be an ordered Banach space. Consider the following conditions.

(i) $\Vert \cdot \Vert$ is absolutely dominating.
(ii) If $a_1,a_2,\ldots \in D^+$, $\sum _{n\in \mathbb {N}}\Vert a_n\Vert <\infty$, then there exist $a\in D^+$, $\varepsilon _n \in (0,\infty )$ with $\varepsilon _n \rightarrow 0$ such that $a_n \le \varepsilon _n a$ for all *n*.^4^
(iii) If $x_1,x_2,\ldots \in D, \sum _{n\in \mathbb {N}}\Vert x_n\Vert <\infty$, then $x_n \xrightarrow {u}0.$

If $D^+$ is closed, then *D* satisfies (ii).

Suppose *D* is Archimedean and directed. Then the following are equivalent.

- $$D^+$$ is closed.
- *D* satisfies (i) and (ii).
- *D* satisfies (iii)

### Proof

Suppose $D^+$ is closed. Let $a_1,a_2,\ldots \in D^+$, $\sum _{n\in \mathbb {N}}\Vert a_n\Vert <\infty$. Choose $\varepsilon _n \in (0,\infty )$, $\varepsilon _n \rightarrow 0$ such that $\sum _{n\in \mathbb {N}}\varepsilon _n^{-1} \Vert a_n\Vert <\infty$. By norm completeness, $a:= \sum _{n\in \mathbb {N}}\varepsilon _n^{-1} a_n$ exists. Because $D^+$ is closed $a \ge \varepsilon _n^{-1} a_n$ for all *n*.

(a)$\Longrightarrow$(b). (ii) is implied by the above argument. By Theorem 3.3 we have (i).

(b)$\Longrightarrow$(c). Let $x_1,x_2,\ldots \in D$, $\sum _{n\in \mathbb {N}}\Vert x_n\Vert <\infty$. Using (i) let $a_1,a_2,\ldots \in D^+$ with $\sum _{n\in \mathbb {N}}\Vert a_n\Vert <\infty$ be such that $-a_n \le x_n \le a_n$. By (ii) it then follows that $x_n \xrightarrow {u}0$.

(c)$\Longrightarrow$(a). Take *b* in the closure of $D^+$. For $n\in \mathbb {N}$, choose $x_n\in D^+$, $\sum _{n\in \mathbb {N}}\Vert x_n - b\Vert <\infty$. By (iii) there exist $a\in D^+$, $\varepsilon _n \in (0,\infty )$ with $\varepsilon _n \rightarrow 0$ and $-\varepsilon _n a \le x_n - b \le \varepsilon _n a$, so that $b \ge x_n - \varepsilon _n a \ge - \varepsilon _n a$. Then $b\ge 0$ because *D* is Archimedean. $\square$

### Lemma 3.10

Let *D* be an ordered Banach space for which $D^+$ is closed. Then

5 $$\begin{aligned} x_1,x_2,\ldots \in D, \ a\in D, \ b\in D, \ \Vert x_n - a\Vert \rightarrow 0, \ x_n \xrightarrow {u}b \quad \Longrightarrow \quad a=b. \end{aligned}$$

### Proof

Assume $b=0$. There exist $c\in D^+$, $\varepsilon _n \in (0,\infty )$ with $\varepsilon _n \downarrow 0$ and $-\varepsilon _n c \le x_n \le \varepsilon _n c$. Then $-\varepsilon _N c \le x_n \le \varepsilon _N c$ whenever $n\ge N$. Since $D^+$ is closed, $-\varepsilon _N c \le a \le \varepsilon _N c$ for all *N*, and so, as *D* is Archimedean by Theorem 3.2, $a=0$. $\square$

With this we can easily prove the following theorem. In a recently published book by Aliprantis and Tourky [1]^5^ but also in older Russian papers by Wulich [5] one can find the proof that an order preserving linear map is continuous (see [1, Theorem 2.32] or combine [5, Theorem III.2.2] (which states the result for $D_2=\mathbb {R}$) and [6, Theorem VI.2.1]). Theorem 3.11 is more general in the sense that it states that linear order bounded maps are continuous.

### Theorem 3.11

Let $D_1,D_2$ be ordered Banach spaces. Suppose $D_1^+$ is closed and generating and $D_2^+$ is closed. Let $T: D_1 \rightarrow D_2$ be linear and order bounded. Then *T* is continuous. Consequently, if *T* is an order isomorphism then it is a homeomorphism.

### Proof

Let $x_1,x_2,\dots \in D_1$, $x_n \rightarrow 0$ and suppose $T x_n \rightarrow c$ for some $c\in D_2$. If from this we can prove $c=0$, then by the Closed Graph Theorem *T* will be continuous. We may assume $\sum _{n\in \mathbb {N}}\Vert x_n\Vert <\infty$. Then $x_n \xrightarrow {u}0$ in $D_1$ (Theorem 3.9), so $T x_n \xrightarrow {u}0$ in $D_2$ (Lemma 3.8). Hence, $c=0$ according to Lemma 3.10. $\square$

We present a consequence as has been done for order preserving linear maps in [6, Theorem VI.2.2].

### Corollary 3.12

Let $D_1$ be an ordered Banach space with closed generating cone. Let $D_2$ be an ordered normed vector space with a normal cone. Let $T: D_1 \rightarrow D_2$ be linear and order bounded. Then *T* is continuous.

### Proof

As $D_2^+$ is normal, we have $D_2^{\sim } = D_2'$ (see [1, Corollary 2.27]). Let $\Delta$ be the unit ball in $D_1$. Then for all $\phi \in D_2'$, the map $\phi \circ T$ is an order bounded functional, whence continuous by Theorem 3.11. Thus $\phi \circ T( \Delta )$ is bounded for all $\phi \in D_2'$. By an application of the Principle of Uniform Boundedness (see [7, Corollary III.4.3]), $T(\Delta )$ is norm bounded. $\square$

### Theorem 3.13

[14, Theorem 6.8] Let *D* be an ordered Banach space with closed cone $D^+$. If $f\in D'$ and $f\in D^\sim$, i.e., *f* is linear continuous and order bounded, then *f* is regular, i.e., there are $f_1,f_2\in D^{\sim +}$ with $f= f_1 - f_2$.

By Theorem 3.13 and Theorem 3.11 we derive the following corollary.

### Corollary 3.14

Let *D* be an ordered Banach space with closed generating cone. Then every order bounded linear map $D \rightarrow \mathbb {R}$ is regular, i.e., $D^\sim = D^{\sim +} - D^{\sim +}$.

## The Bochner integral on ordered Banach spaces

**In this section** $\varvec{(X,\mathcal {A},\mu )}$ **is a complete** $\varvec{\sigma }$ **-finite measure space, with** $\varvec{\mu \ne 0}$ **. For more assumptions see** 4.3 **and** 4.7.

We define the integral of simple functions in Definition 4.1 and recall the definition and facts on Bochner integrability in Definitions 4.2 and 4.4. After that, we consider an ordered Banach space *D*. In Theorems 4.5 and 4.6 we describe the order structure of the space ${\mathfrak {B}}_D$ of Bochner integrable functions. In 4.8 we summarise the results of 4.9–4.16, in which we compare closedness and generatingness of the positive cones of *D* and ${\mathfrak {B}}_D$.

### Definition 4.1

Let *E* be a vector space. We say that a function $f: X\rightarrow E$ is *simple* if there exist $N\in \mathbb {N}$, $a_1,\ldots ,a_N\in E$, $A_1,\ldots , A_N\in \mathcal {A}$ with $\mu (A_1),\ldots ,\mu (A_N)<\infty$ for which

6 $$\begin{aligned} f = \sum _{n=1}^N a_n \mathbbm {1}_{A_n}. \end{aligned}$$

The simple functions form a linear subspace *S* of $E^X$, which is a Riesz subspace of $E^X$ in case *E* is a Riesz space. We define $\varphi : S\rightarrow E$ by

7 $$\begin{aligned} \varphi (f) = \sum _{n=1}^N \mu (A_n) a_n, \end{aligned}$$

where $f,N,A_n,a_n$ are as in (6). $\varphi (f)$ is called the *integral* of *f*. We write $S_\mathbb {R}$ for the linear space of simple functions $X\rightarrow \mathbb {R}$.

Definition 4.2 and the facts in 4.4 can be found in Chapter III in the book by Hille and Phillips [9].

### Definition 4.2

Let $(D,\Vert \cdot \Vert )$ be a Banach space. A function $f: X \rightarrow D$ is called *Bochner integrable* whenever there exists a sequence of simple functions $(s_n)_{n\in \mathbb {N}}$ such that $s_n(x) \rightarrow f(x)$ for $\mu$-almost all $x\in X$ and

8 $$\begin{aligned} \int \Vert f(x) -s_n(x)\Vert {{\mathrm{{\mathrm {d}}}}}\mu (x) \rightarrow 0 . \end{aligned}$$

Then the sequence $(\varphi (s_n ) )_{n\in \mathbb {N}}$ converges. Its limit is independent of the choice of the sequence $(s_n)_{n\in \mathbb {N}}$ and is called the *Bochner integral* of *f*.

### 4.3

**For the rest of this section,** $\varvec{D}$ **is a Banach space (with norm** $\varvec{\Vert \cdot \Vert }$)**, and, we write** $\varvec{{\mathfrak {B}}}$ **(or)** $\varvec{{\mathfrak {B}}_D}$ **for the Banach space of classes of Bochner integrable functions** $\varvec{X \rightarrow D}$ **, with norm** $\varvec{\Vert \cdot \Vert _{\mathfrak {B}}}$ **(see** 4.4 **(b)). We write** $\varvec{{\mathfrak {b}}}$ **(or)** $\varvec{{\mathfrak {b}}_D}$ **for the Bochner integral on** $\varvec{{\mathfrak {B}}}$ **.**

### 4.4

Some facts on the Bochner integrable functions:

- [9, Theorem 3.7.4] and [17, Proposition 2.15] If $f: X \rightarrow D$ is Borel measurable, *f*(*X*) is separable and $\int \Vert f\Vert {{\mathrm{{\mathrm {d}}}}}\mu <\infty$, then *f* is Bochner integrable.
- [9, Theorem 3.7.8] ${\mathfrak {B}}$ is a Banach space under the norm $\Vert \cdot \Vert _{\mathfrak {B}}: {\mathfrak {B}}\rightarrow [0,\infty )$ given by
  9 $$\begin{aligned} \Vert f\Vert _{\mathfrak {B}}= \int \Vert f\Vert {{\mathrm{{\mathrm {d}}}}}\mu . \end{aligned}$$
- [9, Theorem 3.7.12] Let *E* be a Banach space and $T: D \rightarrow E$ be linear and continuous. If $f\in {\mathfrak {B}}_D$, then $T\circ f \in {\mathfrak {B}}_E$ and
  10 $$\begin{aligned} T( {\mathfrak {b}}_D(f)) = {\mathfrak {b}}_E ( T \circ f ). \end{aligned}$$
- [9, Theorem 3.7.9] Let $f_1,f_2,\ldots$ be in ${\mathfrak {B}}$, $f: X \rightarrow D$ and $h \in \mathcal {L}^1(\mu )^+$. Suppose that $f_n(x) \rightarrow f(x)$ and $\Vert f_n(x)\Vert \le h(x)$ for $\mu$-almost all $x\in X$. Then $f \in {\mathfrak {B}}$ and
  11 $$\begin{aligned} {\mathfrak {b}}(f_n) \rightarrow {\mathfrak {b}}(f). \end{aligned}$$
- If *D* is an ordered Banach space then ${\mathfrak {B}}$ is an ordered Banach space under the ordering given by
  12 $$\begin{aligned} f \le g \quad (\hbox {in } {\mathfrak {B}}) \iff f \le g \quad \mu -\text{ a.e. }. \end{aligned}$$

### Theorem 4.5

Let *D* be a Banach lattice. Then ${\mathfrak {B}}_D$ is a Banach lattice and ${\mathfrak {b}}$ is linear and order preserving.

### Proof

The Bochner integrable functions form a Riesz space because of the inequality $\Vert |x| - |y| \Vert \le \Vert x-y\Vert$.

### Theorem 4.6

Let *D* be an ordered Banach space for which $D^+$ is closed. Then ${\mathfrak {b}}$ is order preserving.

### Proof

Let $f\in {\mathfrak {B}}$ and $f\ge 0$. Then

13 $$\begin{aligned} \alpha ( {\mathfrak {b}}(f) ) = \int \alpha \circ f {{\mathrm{{\mathrm {d}}}}}\mu \ge 0 \quad (\alpha \in (D')^+). \end{aligned}$$

Whence ${\mathfrak {b}}(f) \ge 0$, by Theorem 3.2. $\square$

### 4.7

**For the rest of this section,** $\varvec{D}$ **is an ordered Banach space.**

### 4.8

The following is a list of results presented in 4.9–4.16.

- ${\mathfrak {B}}^+$ is closed if and only if $D^+$ is (4.9).
- If ${\mathfrak {B}}$ is directed, then so is *D* (straightforward, see also Theorem 4.12).
- Let $C>0$ and $D^+$ be closed and generating. If $\Vert \cdot \Vert$ is *C*-absolutely dominating, then so is $\Vert \cdot \Vert _{\mathfrak {B}}$ (Lemma 4.11).
- Let $C>0$. If ${\mathfrak {B}}$ is directed and $\Vert \cdot \Vert _{\mathfrak {B}}$ is *C*-absolutely dominating, then so is $\Vert \cdot \Vert$ (Theorem 4.12).
- ${\mathfrak {B}}^+$ is closed and generating if and only if $D^+$ is closed and generating (Theorem 4.16).
- If there exist disjoint $A_1,A_2,\ldots$ in $\mathcal {A}$ with $0< \mu (A) <\infty$ for all *n*: If ${\mathfrak {B}}^+$ is generating, then so is $D^+$ and $\Vert \cdot \Vert$ is absolutely dominating (Corollary 4.14).
- If no such $A_1,A_2,\ldots$ exist: ${\mathfrak {B}}^+$ is generating if and only if $D^+$ is generating (4.15).

### 4.9

Whenever $f\in {\mathfrak {B}}$, $f_n \in {\mathfrak {B}}^+$ with $\int \Vert f-f_n\Vert {{\mathrm{{\mathrm {d}}}}}\mu \rightarrow 0$, then there exist Bochner integrable $g_n\ge 0$ with $\sum _{n\in \mathbb {N}}\int \Vert f-g_n\Vert {{\mathrm{{\mathrm {d}}}}}\mu <\infty$; this implies $g_n \rightarrow f$ $\mu$-almost everywhere. So whenever $D^+$ is closed this implies $f\ge 0$ $\mu$-almost everywhere.

We infer that ${\mathfrak {B}}^+$ is closed whenever $D^+$ is.

On the other hand, if ${\mathfrak {B}}^+$ is closed then so is $D^+$. Indeed, let $A\in \mathcal {A}$, $0<\mu (A)<\infty$. If $a_n\in D^+$ and $a_n \rightarrow a$, then $\Vert a\mathbbm {1}_A - a_n \mathbbm {1}_A \Vert _{{\mathfrak {B}}} \rightarrow 0$. Therefore $a\in D^+$.

### Lemma 4.10

Suppose $D^+$ is generating and $C>0$ is such that $\Vert \cdot \Vert$ is *C*-absolutely dominating. Let $f: X \rightarrow D$ be simple, let $\varepsilon >0$. Then there exists a simple $g: X \rightarrow D^+$ with $-g \le f \le g$ and $\int \Vert g\Vert {{\mathrm{{\mathrm {d}}}}}\mu \le C \int \Vert f\Vert {{\mathrm{{\mathrm {d}}}}}\mu + \varepsilon$.

### Proof

Write $f = \sum \nolimits _{n=1}^N x_n \mathbbm {1}_{A_n}$ with disjoint sets $A_1,\ldots , A_N\in \mathcal {A}$ of finite measure and $x_1,\ldots , x_N\in D$. Let $\kappa = \mu (\bigcup _{n=1}^N A_n)$ and assume $\kappa >0$. For each *n*, choose $a_n\in D^+$, $-a_n \le x_n \le a_n$, $\Vert a_n\Vert \le C\Vert x_n\Vert + \frac{\varepsilon }{\kappa }$. Put $g = \sum \nolimits _{n=1}^N a_n \mathbbm {1}_{A_n}$. Then $-g \le f \le g$ and $\int \Vert g\Vert {{\mathrm{{\mathrm {d}}}}}\mu = \sum \nolimits _{n=1}^N \Vert a_n\Vert \mu (A_n) \le \sum \nolimits _{n=1}^N (C\Vert x_n\Vert +\frac{\varepsilon }{\kappa }) \mu (A_n) = C\int \Vert f\Vert {{\mathrm{{\mathrm {d}}}}}\mu + \varepsilon$. $\square$

### Lemma 4.11

Suppose $D^+$ is closed and generating and $C>0$. Then ${\mathfrak {B}}^+$ is closed and generating. If $\Vert \cdot \Vert$ is *C*-absolutely dominating, then so is $\Vert \cdot \Vert _{\mathfrak {B}}$.

### Proof

${\mathfrak {B}}^+$ is closed by 4.9. Assume that $\Vert \cdot \Vert$ is *C*-absolutely dominating. Let $f: X \rightarrow D$ be Bochner integrable and let $\varepsilon >0$. We prove there exists a Bochner integrable $g: X \rightarrow D^+$ with $-g \le f \le g$ $\mu$-a.e. and $\int \Vert g\Vert {{\mathrm{{\mathrm {d}}}}}\mu \le C (\int \Vert f\Vert {{\mathrm{{\mathrm {d}}}}}\mu +\varepsilon )$. Choose simple functions $s_1,s_2,\ldots$ with $\int \Vert f-s_n\Vert {{\mathrm{{\mathrm {d}}}}}\mu < \varepsilon 2^{-n-1}$. Then $s_n \rightarrow f$ pointwise outside a $\mu$-null set *Y*.

Define $f_1 = s_1$, $f_n = s_n - s_{n-1}$ for $n\in \{2,3,\ldots \}$. Observe

14 $$\begin{aligned} \int \Vert f_1\Vert {{\mathrm{{\mathrm {d}}}}}\mu< & {} \int \Vert f\Vert {{\mathrm{{\mathrm {d}}}}}\mu + \varepsilon 2^{-2}, \nonumber \\ \int \Vert f_n\Vert {{\mathrm{{\mathrm {d}}}}}\mu< & {} \varepsilon 2^{-n-1} + \varepsilon 2^{-n-2} < \varepsilon 2^{-n} \quad (n\in \{2,3,\ldots \}). \end{aligned}$$

For each *n*, choose a simple $g_n : X \rightarrow D^+$ with $-g_n \le f_n \le g_n$ such that

15 $$\begin{aligned} \int \Vert g_1\Vert {{\mathrm{{\mathrm {d}}}}}\mu< & {} C \left( \int \Vert f\Vert {{\mathrm{{\mathrm {d}}}}}\mu + \varepsilon 2^{-2} \right) , \nonumber \\ \int \Vert g_n\Vert {{\mathrm{{\mathrm {d}}}}}\mu< & {} C\varepsilon 2^{-n} \quad (n\in \{2,3,\ldots \}). \end{aligned}$$

As $\sum _{n\in \mathbb {N}}\int \Vert g_n\Vert {{\mathrm{{\mathrm {d}}}}}\mu <\infty$ there is a $\mu$-null set $Z\subset X$ for which

16 $$\begin{aligned} \sum _{n\in \mathbb {N}}\Vert g_n(x)\Vert <\infty \quad (x\in X {\setminus } Z). \end{aligned}$$

Put $X_0 := X {\setminus } (Y\cup Z)$. Define $g: X \rightarrow D^+$ by

17 $$\begin{aligned} g(x) = {\left\{ \begin{array}{ll} \sum _{n\in \mathbb {N}}g_n(x) &{} x\in X_0, \\ 0 &{} x\notin X_0. \end{array}\right. } \end{aligned}$$

Then *g* is Bochner integrable and $\int \Vert g\Vert {{\mathrm{{\mathrm {d}}}}}\mu \le \sum _{n\in \mathbb {N}}\int \Vert g_n\Vert {{\mathrm{{\mathrm {d}}}}}\mu \le C(\int \Vert f\Vert {{\mathrm{{\mathrm {d}}}}}\mu + \varepsilon )$.

Moreover, for $x\in X_0$ we have $-g(x) \le \sum _{n=1}^N f_n(x) = s_N(x) \le g(x)$ for all *N*, whereas $s_N(x) \rightarrow f(x)$ since $x\notin Y$. From the closedness of $D^+$ it follows that $-g \le f \le g$ on $X_0$. $\square$

In the following Theorems (4.12, 4.13, 4.14 and 4.15) we derive properties of *D* from properties of ${\mathfrak {B}}$. In Theorem 4.16 we show that *D* has a closed and generating cone if and only if ${\mathfrak {B}}$ does.

### Theorem 4.12

Assume ${\mathfrak {B}}$ is directed. Let $C>0$ and suppose $\Vert \cdot \Vert _{{\mathfrak {B}}}$ is *C*-absolutely dominating. Then *D* is directed and $\Vert \cdot \Vert$ is *C*-absolutely dominating.

### Proof

Let $x\in D$ and $x\ne 0$. Let $A\in \mathcal {A}$ with $0<\mu (A) <\infty$. Then $x\mathbbm {1}_A\in {\mathfrak {B}}$, $\Vert x\mathbbm {1}_A\Vert _{{\mathfrak {B}}} = \mu (A) \Vert x\Vert$. Let $C'>C$. There is a $g\in {\mathfrak {B}}$ with $-g \le x\mathbbm {1}_A \le g$, $\int \Vert g(t)\Vert {{\mathrm{{\mathrm {d}}}}}\mu (t) \le C' \Vert x\mathbbm {1}_A\Vert _{{\mathfrak {B}}}$. Then $\int _A \Vert g(t)\Vert {{\mathrm{{\mathrm {d}}}}}\mu (t) \le C' \mu (A) \Vert x\Vert$, so $\mu \{t\in A : \Vert g(t)\Vert > C' \Vert x\Vert \} < \mu (A)$. In particular, there is a $t\in A$ with $\Vert g(t)\Vert \le C' \Vert x\Vert$ and $-g(t) \le x \le g(t)$. $\square$

### Theorem 4.13

Let *D* be an ordered Banach space such that the Bochner integrable functions $\mathbb {N}\rightarrow D$ form a directed space. Then *D* is directed and $\Vert \cdot \Vert$ is absolutely dominating.

### Proof

*D* is directed. In case $\Vert \cdot \Vert$ is not absolutely dominating, there exist $x_1,x_2,\ldots \in D$ such that for every *n*

$$\begin{aligned}&2^n \Vert x_n\Vert \le \inf \{ \Vert a\Vert : a\in D^+, - a \le x_n \le a\} \end{aligned}$$

and $\Vert x_n\Vert = 2^{-n}$. Then $n\mapsto x_n$ is Bochner integrable, so, by our assumption, there exist $a_n \in D^+$ with $-a_n \le x_n \le a_n$ for all *n* and $\sum _{n\in \mathbb {N}}\Vert a_n\Vert <\infty$ which is false. $\square$

### Corollary 4.14

Suppose there exist disjoint $A_1,A_2,\ldots$ in $\mathcal {A}$ with $0<\mu (A_n)<\infty$ for all *n*. Suppose ${\mathfrak {B}}$ is directed. Then *D* is directed and $\Vert \cdot \Vert$ is absolutely dominating.

### Proof

This follows from Theorem 4.13 since $f\mapsto \sum _{n\in \mathbb {N}}f(n) \mathbbm {1}_{A_n}$ forms an isometric order preserving isomorphism from the Bochner integrable functions $\mathbb {N}\rightarrow D$ into ${\mathfrak {B}}$. $\square$

### 4.15

Whenever there do not exist $A_1,A_2,\ldots$ as in Corollary 4.14, then $X= A_1 \cup \cdots \cup A_N$, where $A_1,\dots , A_N$ are disjoint atoms. Let $\alpha _n = \mu (A_n) \in (0,\infty )$. Define a norm $\Vert \cdot \Vert _N$ on $D^N$ by $\Vert x\Vert _N = \sum \nolimits _{n=1}^N \Vert x_n\Vert$. Then $T: {\mathfrak {B}}\rightarrow D^N$ defined by $T(\sum \nolimits _{n=1}^N x_n \mathbbm {1}_{A_n}) = (\alpha _1 x_1, \ldots , \alpha _N x_N)$, is an isometric order preserving isomorphism. Therefore,

18 $$\begin{aligned} D^+ \text{ is } \text{ generating } \iff (D^N)^+ \text{ is } \text{ generating } \iff {\mathfrak {B}}^+ \text{ is } \text{ generating }. \end{aligned}$$

### Theorem 4.16

$D^+$ is closed and generating if and only if ${\mathfrak {B}}^+$ is. Moreover, if $D^+$ is closed and generating and $C>0$, then $\Vert \cdot \Vert$ is *C*-absolutely dominating if and only if $\Vert \cdot \Vert _{{\mathfrak {B}}}$ is.

### Proof

This follows from Lemma 4.11 and Theorem 4.12. $\square$

## An extension of the Bochner integral

We present the definition of a Banach cover (Definition 5.2), some examples, and use this notion to extend the Bochner integral to functions with values in such a vector space (5.8).

The next result follows by definition of the Bochner integral or by 4.4(c).

### Theorem 5.1

Let $D_1$ and *D* be Banach spaces and suppose $D_1\subset D$ and the inclusion map is continuous. Suppose $f: X \rightarrow D$ has values in $D_1$ and is Bochner integrable as map $X\rightarrow D_1$ with integral *I*. Then *f* is Bochner integrable as a map $X \rightarrow D$ with integral *I*.

### Definition 5.2

Let *E* be a vector space. Suppose $\mathbb {B}$ is a collection of Banach spaces whose underlying vector spaces are linear subspaces of *E*. $\mathbb {B}$ is called a *Banach cover* of *E* if $\bigcup \mathbb {B}= E$ and for all $D_1$ and $D_2$ in $\mathbb {B}$ there exists a $D\in \mathbb {B}$ with $D_1, D_2\subset D$ such that both inclusion maps $D_1\rightarrow D$ and $D_2 \rightarrow D$ are continuous.

For a $D\in \mathbb {B}$, we write $\Vert \cdot \Vert _D$ for its norm if not indicated otherwise.

If *E* is an ordered vector space, then an *ordered Banach cover* is a Banach cover whose elements are seen as ordered subspaces of *E*.

### 5.3

A bit of pedantry: strictly speaking, a Banach space is a couple $(D,\Vert \cdot \Vert )$ consisting of a vector space *D* and a norm $\Vert \cdot \Vert$. One usually talks about the “Banach space *D*”, the norm being understood. Mostly, we adopt that convention but not always. In the context of the above definitions one has to be careful. A Banach cover may contain several Banach spaces with the same underlying vector space, so that a formula like “$D_1,D_2 \subset D$” really is ambiguous. What we mean is only an inclusion relation between the vector spaces and no connection between the norms is assumed a proiri.

However, suppose $(D_1,\Vert \cdot \Vert _1)$ and $(D_2,\Vert \cdot \Vert _2)$ are elements of a Banach cover $\mathbb {B}$ and $D_1=D_2$. There is a Banach space $(D,\Vert \cdot \Vert _D)$ in $\mathbb {B}$ with $D_1, D_2 \subset D$ and with continuous inclusion maps. If a sequence $(x_n)_{n\in \mathbb {N}}$ in $D_1 (=D_2)$ is $\Vert \cdot \Vert _1$-convergent to *a* and $\Vert \cdot \Vert _2$-convergent to *b*, then it is $\Vert \cdot \Vert _D$-convergent to *a* and *b*, so $a=b$. Hence, by the Closed Graph Theorem the norms $\Vert \cdot \Vert _1$ and $\Vert \cdot \Vert _2$ are equivalent.

Similarly, if $(D_1,\Vert \cdot \Vert _1)$ and $(D_2,\Vert \cdot \Vert _2)$ are elements of a Banach cover and $D_1 \subset D_2$, then the inclusion map $D_1 \rightarrow D_2$ automatically is $\Vert \cdot \Vert _1$-$\Vert \cdot \Vert _2$-continuous.

### Example 5.4

In [18], Thomas considers quasicomplete Hausdorff locally convex vector spaces. For every bounded closed absolutely convex subset *B* of such a space *E* its linear hull $E_B$ is a Banach space under a norm whose closed unit ball is *B*. If $B_1$ and $B_2$ are bounded closed absolutely convex sets, then so is the closure of $B_1+B_2$. It follows that the spaces $E_B$ form a Banach cover of *E*.

### Example 5.5

Let *E* be a uniformly complete Riesz space. The set of principal ideals $\mathbb {B}=\{(E_u,\Vert \cdot \Vert _u): u\in E^+, u \ne 0\}$ is an ordered Banach cover of *E*: for $u,v \in E^+$ with $u,v>0$ one has $E_u, E_v \subset E_{u+v}$ and $\Vert \cdot \Vert _{u+v}\le \Vert \cdot \Vert _u$ on $E_u$.

### Example 5.6

Let $(Y,\mathcal {B},\nu )$ be a complete $\sigma$-finite measure space. Let *M* be the space of classes of measurable functions $Y \rightarrow \mathbb {R}$. A function $\rho : M\rightarrow [0,\infty ]$ is called an *function norm* if (i) $\rho (f) =0 \iff f=0$ a.e., (ii) $\rho (\alpha f) = |\alpha | \rho (f)$ (where $0 \cdot \infty =0$), (iii) $\rho (|f|) =\rho (f)$, (iv) $\rho (f+g) \le \rho (f) + \rho (g)$, (v) $0\le f \le g$ a.e. implies $\rho (f) \le \rho (g)$ for $f,g\in M$ and $\alpha \in \mathbb {R}$. For such function norm $\rho$ the set

19 $$\begin{aligned} L_\rho = \{ f\in M : \rho (f) <\infty \} \end{aligned}$$

is a normed Riesz space called a *Köthe space* (see [11, Ch. III §18] or [12, Ch. 1 §9]).

A Köthe space $L_\rho$ is complete, i.e., a Banach lattice if and only if for all $u_n \in L_\rho ^+$ with $\sum _{n\in \mathbb {N}}\rho (u_n) <\infty$, $\sum _{n\in \mathbb {N}}u_n$ is an element of $L_\rho$ ([11, Theorem 19.3]). Examples of complete Köthe spaces are Orlicz spaces ([11, §20]). In particular, the Banach spaces $L^p(\nu )$ for $p \ge 1$ are Köthe spaces. We introduce other examples:

For $w\in M^+$ with $w>0$ a.e. define $\rho _w : M \rightarrow [0,\infty ]$ by

20 $$\begin{aligned} \rho _w(f) := \int |f| w {{\mathrm{{\mathrm {d}}}}}\nu . \end{aligned}$$

Then $\rho _w$ is a function norm. Both the set $\{ L_{\rho _w} : w\in M^+, w>0 \text{ a.e. }\}$ and the set of all complete Köthe spaces are Banach covers of *M* (see Appendix 1).

### Example 5.7

(See Example 5.4.) In the situation of [18], the functions in our set $\mathfrak {U}$ are precisely the functions called “totally summable” by Thomas. Apparently, the totally summable functions form a vector space, and the integral of such a function in the sense of 5.8 equals its Pettis integral as considered by Thomas.

### 5.8

Let *E* be a vector space with a Banach cover $\mathbb {B}$. Let $(X,\mathcal {A},\mu )$ be a complete $\sigma$-finite measure space, $\mu \ne 0$.

1. For $D\subset \mathbb {B}$ denote by $B_D$ the vector space of all Bochner integrable functions $X\rightarrow D$, and, by $b_D$ the Bochner integral $B_D \rightarrow D$.
2. Let $D_1,D_2\in \mathbb {B}$, $f_1 \in B_{D_1}$, $f_2\in B_{D_2}$, $f_1 = f_2$ $\mu$-a.e. Then $b_{D_1} (f_1) = b_{D_2}(f_2)$. *Proof.* Choose $D\in \mathbb {B}$ as in Definition 5.2. Then $f_1,f_2\in B_D$ and $b_{D_1}(f_1) = b_D (f_1) = b_D (f_2) = b_{D_2}(f_2)$.
3. If $D_1,D_2\in \mathbb {B}$ have the same underlying vector space, then $B_{D_1}=B_{D_2}$ since the identity map $D_1 \rightarrow D_2$ is a homeomorphism (see 5.3).
4. We call a function $f: X \rightarrow E$ $\mathbb {B}$-*integrable* if there is a $D\in \mathbb {B}$ such that *f* is $\mu$-a.e. equal to some element of $B_D$.
5. By $\mathfrak {U}$ we indicate the vector space of all $\mu$-equivalence classes of $\mathbb {B}$-integrable functions.

For $D\in \mathbb {B}$ we have a natural map $T_D: B_D \rightarrow \mathfrak {U}$, assigning to every element of $B_D$ its $\mu$-equivalence class. The space $T_D(B_D)$ is a Banach space, which we indicate by ${\mathfrak {B}}_D$.^6^ We write ${\mathfrak {b}}_D$ for the map ${\mathfrak {B}}_D \rightarrow D$ determined by

21 $$\begin{aligned} {\mathfrak {b}}_D (T_D(f)) = b_D(f) \quad (f\in B_D). \end{aligned}$$

$\mathfrak {U}$ is the union of the sets ${\mathfrak {B}}_D$. By (2) there is a unique $\mathfrak {u}: \mathfrak {U}\rightarrow E$ determined by

22 $$\begin{aligned} \mathfrak {u}( f) = {\mathfrak {b}}_D(f) \quad (D\in \mathbb {B}, f\in {\mathfrak {B}}_D). \end{aligned}$$

The above leads to the following theorem.

### Theorem 5.9

$\mathfrak {U}$ is a vector space, $\mathfrak {u}$ is linear and $\{{\mathfrak {B}}_D: D\in \mathbb {B}\}$ is a Banach cover of $\mathfrak {U}$.

### 5.10

1. For any vector space the finite dimensional linear subspaces form a Banach cover.
2. If *E* is a Banach space, then $\{E\}$ is a Banach cover.
3. If *E* is a Banach space and $\mathbb {B}$ is a Banach cover with $E\in \mathbb {B}$, then $\mathfrak {U}$ is just the space of (classes of) Bochner integrable functions $X \rightarrow E$ and $\mathfrak {u}$ is the Bochner integral.
4. A special case of (3): Let *E* be a uniformly complete Riesz space with a unit *e* and let $\mathbb {B}$ be the Banach cover of principal ideals as in Example 5.5. Then $(E,\Vert \cdot \Vert _e)\in \mathbb {B}$.

### 5.11

Let *E* be a vector space and $\mathbb {B}_1$ and $\mathbb {B}_2$ be Banach covers of *E*. Suppose that for all $D_1 \in \mathbb {B}_1$ there exists a $D_2 \in \mathbb {B}_2$ with $D_1 \subset D_2$ such that the inclusion map is continuous. Write $\mathfrak {U}_i, \mathfrak {u}_i$ for the set of $\mathbb {B}_i$-integrable functions and the $\mathbb {B}_i$-integral, for $i\in \{1,2\}$. Then $\mathfrak {U}_1 \subset \mathfrak {U}_2$ and $\mathfrak {u}_1 = \mathfrak {u}_2$ on $\mathfrak {U}_1$.

### Example 5.12

(Different covers and different integrals) Let $(E,\Vert \cdot \Vert )$ be an infinite dimensional Banach space. Let $T: E \rightarrow E$ a linear bijection that is not continuous; say, there exist $x_n \in E$ with $\Vert x_n\Vert = 2^{-n}$, $Tx_n = x_n$, $T(\sum _{n\in \mathbb {N}}x_n) \ne \sum _{n\in \mathbb {N}}x_n$. Define $\Vert x\Vert _T = \Vert Tx\Vert$ for $x\in E$. Then $(E,\Vert \cdot \Vert _T)$ is a Banach space. The map $f:\mathbb {N}\rightarrow E$ given by $f(n)= x_n$ is Bochner integrable in $(E,\Vert \cdot \Vert )$ and in $(E,\Vert \cdot \Vert _T)$, but the integrals do not agree. Whence with $\mathbb {B}_1 = \{ (E,\Vert \cdot \Vert )\}$ and $\mathbb {B}_2 = \{ (E,\Vert \cdot \Vert _T)\}$ we have $f\in \mathfrak {U}_1 \cap \mathfrak {U}_2$ but $\mathfrak {u}_1(f) \ne \mathfrak {u}_2(f)$.

As an immediate consequence of Theorem 4.5 we obtain the following theorem.

### Theorem 5.13

Suppose *E* is a Riesz space and $\mathbb {B}$ is a Banach cover of *E* that consists of Banach lattices that are Riesz subspaces of *E*. Then $\mathfrak {U}$ is a Riesz space and $\mathfrak {u}$ is order preserving.

### Proof

This is a consequence of Theorem 4.5. $\square$

### 5.14

Whenever *E* is an ordered vector space and $\mathbb {B}$ an ordered Banach cover of *E*, then $\mathfrak {U}$ is an ordered vector space. In order for $\mathfrak {u}$ to be order preserving, one needs a condition on $\mathbb {B}$. This and other matters will be treated in Sect. 7. A sufficient condition turns out to be closedness of $D^+$ for every $D\in \mathbb {B}$ (see Theorems 4.6 and 7.1). First we will see in Sect. 6 that all Archimedean directed ordered vector spaces admit such ordered Banach covers. (The Archimedean property is necessary as follows easily from Theorem 3.2).

### 5.15

Whenever *E* is a vector space and $\mathbb {B}$ is a Banach cover of *E*, then the set

23 $$\begin{aligned} \{ A\subset E: \text{ there } \text{ exists } \text{ a } D\in \mathbb {B} \text{ such } \text{ that } A \text{ is } \text{ bounded } \text{ in } D\} \end{aligned}$$

forms a bornology on *E* (we refer to the book of Hogbe-Nlend [10] for the theory of bornologies).

## Covers of ordered Banach spaces with closed generating cones

**In this section** $\varvec{E}$ **is an Archimedean directed ordered vector space.** $\varvec{\mathbb {B}}$ **is the collection of all ordered Banach spaces that are ordered linear subspaces of** $\varvec{E}$ **whose cones are closed and generating.**

We intend to prove that $\mathbb {B}$ is a Banach cover of *E* (Theorem 6.5).

### Lemma 6.1

Let $(D_1, \Vert \cdot \Vert _1)$, $(D_2,\Vert \cdot \Vert _2)$ be in $\mathbb {B}$. Let $z_1,z_2,\ldots \in D_1 \cap D_2$, $a\in D_1$, $b\in D_2$, $\Vert z_n - a\Vert _1 \rightarrow 0$, $\Vert z_n - b\Vert _2 \rightarrow 0$. Then $a=b$.

### Proof

We may assume $\sum _{n\in \mathbb {N}}\Vert z_n - a\Vert _1 <\infty$ and $\sum _{n\in \mathbb {N}}\Vert z_n - b\Vert _2 <\infty$. Then $z_n \xrightarrow {u}a$ in $D_1$ and $z_n \xrightarrow {u}b$ in $D_2$ by Theorem 3.9. Then $z_n \xrightarrow {u}a$ and $z_n \xrightarrow {u}b$ in *E*. Because *E* is Archimedean, $a=b$. $\square$

### 6.2

If *D* is an ordered Banach space with closed generating cone $D^+$, under each of two norms $\Vert \cdot \Vert _1$ and $\Vert \cdot \Vert _2$, then these norms are equivalent. Indeed, the identity map $(D,\Vert \cdot \Vert _1) \rightarrow (D,\Vert \cdot \Vert _2)$ has a closed graph by Lemma 6.1.

### Theorem 6.3

Let $(D_1, \Vert \cdot \Vert _1)$, $(D_2,\Vert \cdot \Vert _2)$ be in $\mathbb {B}$. $D_1+D_2$ is an ordered Banach space with closed generating cone under the norm $\Vert \cdot \Vert : D_1 + D_2 \rightarrow [0,\infty )$ defined by

24 $$\begin{aligned} \Vert z\Vert := \inf \{ \Vert x\Vert _1 + \Vert y\Vert _2 : x\in D_1, y\in D_2, z= x+y\}. \end{aligned}$$

Moreover, if $C>0$ and $\Vert \cdot \Vert _1$ and $\Vert \cdot \Vert _2$ are *C*-absolutely dominating, then so is $\Vert \cdot \Vert$.

### Proof

$D_1 \times D_2$ is a Banach space under the norm $(x, y) \mapsto \Vert x\Vert _1 + \Vert y\Vert _2$. From Lemma 6.1 it follows that $\Delta := \{(a,b)\in D_1 \times D_2: a=-b\}$ is closed in $D_1 \times D_2$. Then $D_1 \times D_2 / \Delta$ is a Banach space under the quotient norm. This means that $D_1 + D_2$ is a Banach space under $\Vert \cdot \Vert$. (In particular, $\Vert \cdot \Vert$ is a norm.)

Since $D_1^+ + D_2^+ \subset (D_1+D_2)^+$, the latter is generating.

We prove that $(D_1+D_2)^+$ is closed. Let $u_1,u_2,\ldots \in D_1+D_2$, $\sum _{n\in \mathbb {N}}\Vert u_n\Vert <\infty$; we prove $u_n \xrightarrow {u}0$. Choose $x_n \in D_1$, $y_n\in D_2$ with $u_n = x_n + y_n$ and $\sum _{n\in \mathbb {N}}\Vert x_n\Vert _1 <\infty$, $\sum _{n\in \mathbb {N}}\Vert y_n\Vert _2<\infty$. Then, see Theorem 3.9, $x_n \xrightarrow {u}0$ in $D_1$ and $y_n \xrightarrow {u}0$ in $D_2$. It follows that $u_n \xrightarrow {u}0$ in $D_1+D_2$. By Theorem 3.9 it follows that $(D_1+ D_2)^+$ is closed.

Suppose $C>0$ is such that $\Vert \cdot \Vert _1$ and $\Vert \cdot \Vert _2$ are *C*-absolutely dominating. Let $z\in D_1 + D_2$, $\varepsilon >0$. Choose $x\in D_1$, $y\in D_2$ with $z= x+y$, $\Vert x\Vert _1 + \Vert y\Vert _2 \le \Vert z\Vert + \frac{\varepsilon }{3}$. Choose $a\in D_1^+$ with $-a\le x \le a$, $\Vert a\Vert _1 < C\Vert x\Vert _1 + \frac{\varepsilon }{3}$ and $b\in D_2^+$ with $-b \le y \le b$, $\Vert b\Vert _2< C\Vert y\Vert _2 +\frac{\varepsilon }{3}$. Set $c = a+b$. Then $c\in (D_1+D_2)^+$, $-c\le z\le c$ and $\Vert c\Vert \le \Vert a\Vert _1+ \Vert b\Vert _2< C\Vert x\Vert _1 + C\Vert y\Vert _2 + 2 \frac{\varepsilon }{3} < C\Vert z\Vert +\varepsilon$. $\square$

### 6.4

Let $x\in E$. (We make a $D\in \mathbb {B}$ with $x\in D$.) Choose $a\in E$ such that $-a\le x \le a$. Let $D= \mathbb {R}( a- x) + \mathbb {R}( a+x)= \mathbb {R}a+ \mathbb {R}x$. *D* is a directed ordered vector space. Define $\Vert \cdot \Vert : D \rightarrow [0,\infty )$ by

25 $$\begin{aligned} \Vert y\Vert := \inf \{ s \ge 0 : -sa \le y \le s a\}. \end{aligned}$$

Then $\Vert \cdot \Vert$ is a norm on *D*, $-\Vert y\Vert a \le y \le \Vert y\Vert a$ for all $y\in D$ and $\Vert a\Vert =1$. Thus $(D,\Vert \cdot \Vert )$ is a directed ordered Banach space. Moreover $D^+$ is closed: Let $y\in D$, $y_1,y_2,\dots \in D^+$, $\Vert y -y_n\Vert < \frac{1}{n}$. Then $y \ge y_n - \frac{1}{n} a\ge - \frac{1}{n} a$, so $y \ge 0$.

$\Vert \cdot \Vert$ is 1-absolutely dominating: $\Vert a\Vert =1$, so $\inf \{\Vert c\Vert : c\in D^+, -c \le y \le c\} \le \inf \{ s \ge 0: -sa \le y \le sa\} = \Vert y\Vert$.

Even $\Vert y \Vert = \inf \{\Vert c\Vert : c\in D^+, -c \le y \le c\}$: For $c\in D^+$ with $-c \le y \le c$ and $s\ge 0$ such that $c \le s a$ we have $-sa \le -c \le y \le c \le sa$ and so $\Vert y\Vert \le \Vert c\Vert$.

### Theorem 6.5

$\mathbb {B}$ is a Banach cover of *E*. Moreover,

26 $$\begin{aligned} \{ D\in \mathbb {B}: \Vert \cdot \Vert _D \text{ is }\,\, 1{ -absolutely \, dominating }\} \end{aligned}$$

is a Banach cover of *E*.

### Proof

By 6.4 each element of *E* is contained in an ordered Banach space with closed generating cone (with a 1-absolutely dominating norm). By Theorem 6.3 and by definition of the norm, $\mathbb {B}$ forms a Banach cover of *E*. $\square$

### 6.6

It is reasonable to ask if an analogue of Theorem 6.5 holds in the world of Riesz spaces: does every Archimedean Riesz space have a Banach cover consisting of Riesz spaces? The answer is negative.

Let *E* be the Riesz space of all functions *f* on $\mathbb {N}$ for which there exist $N\in \mathbb {N}$ and $r,s\in \mathbb {R}$ such that $f(n) = sn + r$ for $n\ge N$. Suppose *E* has a Banach cover $\mathbb {B}$ consisting of Riesz subspaces of *E*. There is a $D\in \mathbb {B}$ that contains the constant function $\mathbbm {1}$ and the identity map $i : \mathbb {N}\rightarrow \mathbb {N}$. For every $n\in \mathbb {N}$,

27 $$\begin{aligned} \mathbbm {1}_{\{1,\ldots ,n\}} = \mathbbm {1} \vee (n+1) \mathbbm {1} - i \vee n\mathbbm {1} \in D. \end{aligned}$$

It follows that $D=E$, so *E* is a Banach space under some norm.

But *E* is the union of an increasing sequence $D_1 \subset D_2 \subset \cdots$ of finite dimensional—hence, closed—linear subspaces:

28 $$\begin{aligned} D_n = \mathbb {R}\mathbbm {1} + \mathbb {R}i + \mathbb {R}\mathbbm {1}_{\{1\}} + \cdots + \mathbb {R}\mathbbm {1}_{\{n\}}. \end{aligned}$$

By Baire’s Category Theorem, some $D_n$ has nonempty interior in *E*. Then $E=D_n$ and we have a contradiction.

### 6.7

In Theorem 6.5 we single out one particular Banach cover $\mathbb {B}$. If we consider only Banach covers consisting of directed spaces with closed cones, this $\mathbb {B}$ is the largest and gives us the largest collection of integrable functions. Without directedness there may not be a largest Banach cover. For instance, consider Example 5.12. Impose on *E* the trivial ordering ($x\le y$ if and only if $x=y$). Then $E^+=\{0\}$, and both $\mathbb {B}_1$ and $\mathbb {B}_2$ consist of Banach spaces with closed (but not generating) cones.

## The integral for an Archimedean ordered vector space

As a consequence of Theorem 4.6 we obtain the following extension of Theorem 5.13.

### Theorem 7.1

Let *E* be an ordered vector space with an ordered Banach cover $\mathbb {B}$ so that $D^+$ is closed for all $D\in \mathbb {B}$. Then $\mathfrak {u}$ is order preserving. Moreover, *E* and $\mathfrak {U}$ are Archimedean.

### Lemma 7.2

Let *D* be an ordered Banach space with a closed generating cone $D^+$. Let *T* be a linear order preserving map of *D* into an Archimedean ordered vector space *H*. Then $\ker T$ is closed and *T*(*D*) equipped with the norm $\Vert \cdot \Vert _q$ given by

29 $$\begin{aligned} \Vert z\Vert _q = \inf \{ \Vert x\Vert : x\in D, Tx =z\}, \end{aligned}$$

has a closed generating cone $T(D)^+$.

### Proof

(I) Let $x_1,x_2,\ldots \in \ker T$, $x\in D$, $x_n \rightarrow x$; we prove $x\in \ker T$. We assume $\sum _{n\in \mathbb {N}}\Vert x- x_n\Vert <\infty$. By Theorem 3.9 $x_n \xrightarrow {u} x$. By Lemma 3.8 $Tx_n \xrightarrow {u} Tx$, so $Tx=0$. (II) $D/ \ker T$ is a Banach space under the quotient norm $\Vert \cdot \Vert _Q$. The formula $x+ \ker T \mapsto Tx$ describes a linear bijection $D/\ker T \rightarrow T(D)$ and

30 $$\begin{aligned} \Vert x + \ker T\Vert _Q = \Vert Tx\Vert _q \quad (x\in D). \end{aligned}$$

It follows that $\Vert \cdot \Vert _q$ is indeed a norm, turning *T*(*D*) into a Banach space.

$T(D^+) \subset T(D)^+$, whence *T*(*D*) is directed.

We prove that *T*(*D*) satisfies (iii) of Theorem 3.9: Let $z_1,z_2,\dots \in T(D)$ be such that $\sum _{n\in \mathbb {N}}\Vert z_n\Vert _q<\infty$. Choose $x_n\in D$ such that $T x_n = z_n$, $\sum _{n\in \mathbb {N}}\Vert x_n\Vert <\infty$. Using (iii) for *D*, $x_n \xrightarrow {u}0$. Then $z_n= Tx_n \xrightarrow {u}0$ by Lemma 3.8. $\square$

### 7.3

In the proof of Lemma 7.2 we mentioned the inclusion $T(D^+) \subset T(D)^+$. This inclusion can be strict. Take $D= H=\mathbb {R}^2$, $T(x,y) = (x, x+y)$. Then $T(D^+) \ne T(D)^+$.

From Theorems 3.11, 4.4(c) and Lemma 7.2 we get:

### Theorem 7.4

Let $E_1, E_2$ be ordered vector spaces, $E_i$ endowed with the Banach cover $\mathbb {B}_i$ consisting of the ordered Banach spaces with closed generating cones. Let $T: E_1 \rightarrow E_2$ be linear and order preserving. If $f: X \rightarrow E_1$ is $\mathbb {B}_1$-integrable, then $T\circ f: X \rightarrow E_2$ is $\mathbb {B}_2$-integrable, and $\mathfrak {u}_2( T \circ f ) = T( \mathfrak {u}_1( f ))$.

### 7.5

In view of Theorem 3.11 the reader may wonder why in Theorem 7.4 *T* is required to be order preserving and not just order bounded, the more so because of the following considerations. Let *D* and *H* be as in Lemma 7.2 and *T* be a linear order bounded map of *D* into *H*. As the implication (c) $\Longrightarrow$ (a) of Theorem 3.9 is valid for Archimedean (but not necessarily directed) *D*, following the lines of the proof of Lemma 7.2 $\ker T$ is closed and *T*(*D*) equipped with the norm as in (29) has a closed cone $T(D)^+$. However, we also need *T*(*D*) to be directed and order boundedness of *T* is no guarantee for that.

An alternative approach might be to drop the directedness condition on the spaces that constitute $\mathbb {B}$. However, the ordered Banach spaces with closed cones may not form a Banach cover.

For an example, let *E* be $\ell ^\infty$ and let $\mathbb {B}$ be the collection of all ordered Banach spaces that are subspaces of $\ell ^\infty$ and have closed cones. We make $D_1,D_2\in \mathbb {B}$. For $D_1$ we take $\ell ^\infty$ with the usual norm $\Vert \cdot \Vert _\infty$. Choose a linear bijection $T: \ell ^\infty \rightarrow \ell ^\infty$ that is not continuous. For $a\in \ell ^\infty$ put $a' = (a_1,-a_1,a_2,-a_2,\dots )$. For $D_2$ we take the vector space $\{ a' : a\in \ell ^\infty \}$ with the norm $\Vert \cdot \Vert _T$ given by $\Vert a'\Vert _T= \Vert Ta\Vert _\infty$. Then $D_2$ is a Banach space and $D_2^+$, begin $\{0\}$, is closed in $D_2$. Suppose $\mathbb {B}$ is a Banach cover. Let *D* be as in Definition 5.2. By the Open Mapping Theorem the identity map $D_1 \rightarrow D$ is a homeomorphism. By the continuity of the inclusion map $D_2 \rightarrow D$ there exists a number *c* such that $\Vert a'\Vert _T \le c\Vert a'\Vert _\infty$ for all $a\in \ell ^\infty$. Then $\Vert Ta\Vert _\infty \le c\Vert a'\Vert _\infty \le c\Vert a\Vert _\infty$ for $a\in \ell ^\infty$, so *T* is continuous. Contradiction.

### Theorem 7.6

Let *E* be an ordered vector space such that $E^\sim$ separates the points of *E*.^7^ Assume $\mathbb {B}$ is a Banach cover consisting of ordered Banach spaces with closed generating cones. Let $f\in \mathfrak {U}$. Then $\alpha \circ f \in \mathcal {L}^1(\mu )$ for all $\alpha \in E^\sim$. Moreover, $I\in E$ is such that

31 $$\begin{aligned} \alpha (I) = \int \alpha \circ f {{\mathrm{{\mathrm {d}}}}}\mu \ \text{ for } \text{ all } \ \alpha \in E^\sim , \end{aligned}$$

if and only if $I=\mathfrak {u}(f)$.

### Proof

Let $f\in \mathfrak {U}$ and let $I= \mathfrak {u}(f)$. By Theorem 7.4 $\alpha \circ f \in \mathcal {L}^1(\mu )$ for all $\alpha \in E^\sim$ and (31) holds. $I=\mathfrak {u}(f)$ is the only element of *E* for which (31) holds because $E^\sim$ separates the points of *E*. $\square$

### Remark 7.7

Functions with values in a Banach space that are Bochner integrable are also Pettis integrable. To some extent the statement of Theorem 7.6 is similar. Indeed, the definition of Pettis integrability could be generalised for vector spaces *V* which are equipped with a set *S* of linear maps $V \rightarrow \mathbb {R}$ that separates the points of *V*, in the sense that one calls a function $f: X \rightarrow V$ Pettis integrable if $\alpha \circ f \in \mathcal {L}^1(\mu )$ for all $\alpha \in S$ and there exists a $I\in V$ such that $\alpha (I)= \int \alpha \circ f {{\mathrm{{\mathrm {d}}}}}\mu$ for all $\alpha \in S$. Then Theorem 7.6 implies that every $f\in \mathfrak {U}$ is Pettis integrable when considering $V=E$ and $S=E^\sim$. Observe, however, that even for a Riesz space *E*, $E^\sim$ may be trivial (see, e.g., [11, 5.A]).

### Theorem 7.8

Let *E* be an ordered quasicomplete Hausdorff locally convex vector space with a closed generating cone. Let $\mathbb {B}_1$ be the Banach cover generated by the bounded closed absolutely convex subsets of *E* (see Example 5.4). Let $\mathbb {B}_2$ be the Banach cover of ordered Banach spaces with closed generating cones (see Section 6, *E* is Archimedean by Theorem 3.2). Let $\mathfrak {U}_i$ and $\mathfrak {u}_i$ denote the $\mathbb {B}_i$-integrable functions and $\mathbb {B}_i$-integral, respectively. Then $\mathfrak {u}_1 = \mathfrak {u}_2$ on $\mathfrak {U}_1 \cap \mathfrak {U}_2$.

### Proof

If $f\in \mathfrak {U}_1$, then *f* is Pettis integrable in the sense of Thomas [18, Definition 1] (or in Remark 7.7 with $V=E$ and $S= E'$). By Theorem 7.6, for $f\in \mathfrak {U}_1 \cap \mathfrak {U}_2$

32 $$\begin{aligned} \alpha (\mathfrak {u}_1(f)) = \int \alpha \circ f {{\mathrm{{\mathrm {d}}}}}\!\mu = \alpha ( \mathfrak {u}_2(f)) \qquad (\alpha \in (E')^+), \end{aligned}$$

implying $\mathfrak {u}_1(f) = \mathfrak {u}_2(f)$ by Theorem 3.2. $\square$

## Comparison with other integrals

**In this section** $\varvec{(X,\mathcal {A},\mu )}$ **is a complete** $\varvec{\sigma }$ **-finite measure space and** $\varvec{E}$ **is a directed ordered vector space with an ordered Banach cover** $\varvec{\mathbb {B}}$ **so that** $\varvec{D^+}$ **is closed for each** $\varvec{D\in \mathbb {B}}$ **.**

In 5.8 we have introduced an integral $\mathfrak {u}$ on a space $\mathfrak {U}$ of $\mathbb {B}$-integrable functions $X \rightarrow E$.^8^ In [16], starting from a natural integral $\varphi$ on the space *S* of all simple functions $X\rightarrow E$ we have made integrals $\varphi _V, \varphi _L, \varphi _{LV}, \ldots$ on spaces $S_V, S_L, S_{LV},\ldots$.

There is an elementary connection: *S* is part of $\mathfrak {U}$ and $\mathfrak {u}$ coincides with $\varphi$ on *S*. (Indeed, let $f\in S$. Being a finite set, *f*(*X*) is contained in *D* for some $D\in \mathbb {B}$. Then *f* is Bochner integrable as a map $X\rightarrow D$.)

In general, $S_V$ and $S_L$ are not subsets of $\mathfrak {U}$, but we can prove that $\mathfrak {u}$ coincides with $\varphi _V$ on $S_V \cap \mathfrak {U}$ and with $\varphi _L$ on $S_L \cap \mathfrak {U}$. Better than that: $\mathfrak {u}$ is “compatible” with $\varphi _V$ in the sense that $\mathfrak {u}$ and $\varphi _V$ have a common order preserving linear extension $S_V + \mathfrak {U}\rightarrow E$. Similarly, $\mathfrak {u}$ is “compatible with $\varphi _L$, $\varphi _{LV},\ldots$”.

### Lemma 8.1

- Let $$f\in \mathfrak {U}$$, $$g\in S_{V}$$, $$f\le g$$. Then $$\mathfrak {u}(f) \le \varphi _V(g)$$.
- Let $$f\in \mathfrak {U}$$, $$g\in S_{L}$$, $$f\le g$$. Then $$\mathfrak {u}(f) \le \varphi _L(g)$$.

### Proof

(a) By the definition of $\varphi _V$ and by the text preceding this lemma we have $\varphi _V(g) = \inf \{ \varphi (h) : h\in S, h \ge g \} = \inf \{ \mathfrak {u}(h) : h\in S, h\ge g\}$. As $g \ge f$ and $\mathfrak {u}$ is order preserving (Theorem 7.1), it follows that $\varphi _V(g) \ge \mathfrak {u}(f)$.

(b) Let $g\in S_L$ and assume $f\le g$. Let $g_1,g_2\in S_L^+$ be such that $g= g_1 - g_2$. Let $(B_i)_{i\in \mathbb {N}}$ be a $\varphi$-partition for both $g_1$ and $g_2$. Write $A_n = \bigcup _{i=1}^n B_i$ for $n\in \mathbb {N}$. Then $f\mathbbm {1}_{A_n} \le g\mathbbm {1}_{A_n}$, thus by (a) (and Theorem 7.1)

33 $$\begin{aligned} \mathfrak {u}(f\mathbbm {1}_{A_n}) \nonumber\le & {} \mathfrak {u}(g \mathbbm {1}_{A_n}) = \varphi (g\mathbbm {1}_{A_n}) = \varphi (g_1 \mathbbm {1}_{A_n}) - \varphi (g_2 \mathbbm {1}_{A_n}) \nonumber \\\le & {} \varphi _L(g_1) - \varphi (g_2 \mathbbm {1}_{A_k }) \quad (k\in \mathbb {N}, k<n). \end{aligned}$$

Which implies $\mathfrak {u}(f\mathbbm {1}_{A_n}) + \varphi (g_2\mathbbm {1}_{A_k}) \le \varphi _L(g_1)$ for all $k<n$. Then letting *n* tend to $\infty$ (apply 4.4(d): $f(x)\mathbbm {1}_{A_n}(x) \rightarrow f(x)$ for all $x\in X$) we obtain

34 $$\begin{aligned} \mathfrak {u}(f) \le \varphi _L(g_1) - \varphi (g_2\mathbbm {1}_{A_k}) \quad (k\in \mathbb {N}), \end{aligned}$$

from which we conclude $\mathfrak {u}(f) \le \varphi _L(g)$. $\square$

### Theorem 8.2

- If $g\in S_{LV}$ and $f\le g$, then $\mathfrak {u}(f) \le \varphi _{LV}(g)$.
- If $S_V$ is stable, $g\in S_{VLV}$ and $f\le g$, then $\mathfrak {u}(f) \le \varphi _{VLV}(g)$.

### Proof

Follow the lines of the proof of the lemma with $S_V$, $S_L$ or $S_{VL}$ instead of *S*. $\square$

### 8.3

1. The theorem supersedes the lemma because $S_V + S_L \subset S_{LV}$.
2. As a consequence, $\mathfrak {u}= \varphi _{LV}$ on $\mathfrak {U}\cap S_{LV}$, and $\mathfrak {u}= \varphi _{VLV}$ on $\mathfrak {U}\cap S_{VLV}$ if $S_V$ is stable.
3. Recall that stability of $S_V$ is necessary for the existence of $S_{VLV}$.

### Theorem 8.4

Let *E* be a uniformly complete Riesz space and $\mathbb {B}$ be the Banach cover of principal ideals (see Example 5.5). $\mathfrak {U}$ is a linear subspace of $S_{LV}$ and $\mathfrak {u}= \varphi _{LV}$ on $\mathfrak {U}$.

### Proof

Let $u\in E^+$ and let $f: X\rightarrow E_u$ be Bochner integrable. We prove $f\in S_{LV}$.

For simplicity of notation, put $D=E_u$. Let $S^D$ be the space of simple functions $X\rightarrow D$. By [16, Corollary 9.8] we have $f\in (S^D)_{LV}$ and $\mathfrak {u}(f) = \varphi _{LV}(f)$. Since *D* is a Riesz ideal in *E*, the identity map $D\rightarrow E$ is order continuous. Then [16, Theorem 8.14] implies $f\in S_{LV}$. $\square$

Contrary to Theorem 8.4, in [16, Example 9.9(II)] $\mathfrak {U}$ is not a linear subspace of $S_{LV}$. Then next example shows, in the context of Theorem 8.4, that the inclusion may be strict.

### Example 8.5

($\mathfrak {U}\subsetneq S_{LV}$) For $X=\mathbb {N}, \mathcal {A}= \mathcal {P}(\mathbb {N})$ and $\mu$ the counting measure and $E=c$. As is mentioned in [16, Examples 9.9(I)], the function $n\mapsto \mathbbm {1}_{\{n\}}$ is an element of $S_{LV}$ but not Bochner integrable. With $\mathbb {B}$ the Banach cover of principal ideals, the function $n \mapsto \mathbbm {1}_{\{n\}}$ is not $\mathbb {B}$-integrable (see 5.10(4)).

## An example: convolution

To illustrate the $\mathbb {B}$-integral as an extension of the Bochner integral we consider the following situation. (This introduction requires some knowledge of harmonic analysis on locally compact groups, the balance of this section does not.)

Let *G* be a locally compact group. For $f: G\rightarrow \mathbb {R}$ and $x\in G$ we let $L_x f : G \rightarrow \mathbb {R}$ be the function $y\mapsto f(x^{-1}y)$.

For a finite measure $\mu$ on *G* and *f* in $L^1(G)$ one defines their convolution product to be the element $\mu * f$ of $L^1(G)$ given for almost every $y\in G$ by

35 $$\begin{aligned} (\mu * f)(y) = \int f(x^{-1} y ) {{\mathrm{{\mathrm {d}}}}}\mu (x) = \int (L_x f)(y) {{\mathrm{{\mathrm {d}}}}}\mu (x). \end{aligned}$$

The map $x\mapsto L_x f$ of *G* into $L^1(G)$ is continuous and bounded, hence Bochner integrable with respect to $\mu$. It is not very difficult to prove that

36 $$\begin{aligned} \mu * f = \int L_x f {{\mathrm{{\mathrm {d}}}}}\mu (x). \end{aligned}$$

Similar statements are true for other spaces of functions instead of $L^1(G)$, such as $L^p(G)$, with $1<p<\infty$, and $C_0(G)$, the space of continuous functions that vanish at infinity.

But consider the space *C*(*G*) of all continuous functions on *G*. The integrals $\int f(x^{-1}y) {{\mathrm{{\mathrm {d}}}}}\mu (x)$ will not exist for all $f\in C(G)$, $y\in G$ and all finite measures $\mu$, but they do if $\mu$ has compact support. Thus, one can reasonably define $\mu * f$ for $f\in C(G)$ and compactly supported $\mu$. However, there is no natural norm on *C*(*G*) (except, of course, if *G* is compact), so we cannot speak of $\int L_x f {{\mathrm{{\mathrm {d}}}}}\mu (x)$ as a Bochner integral. We will see that, at least for $\sigma$-compact *G*, it is a $\mathbb {B}$-integral where $\mathbb {B}$ is the Banach cover of *C*(*G*) that consists of the principal (Riesz) ideals.

### Theorem 9.1

Let *G* be a $\sigma$-compact locally compact group. For every $f\in C(G)$ there exists a $w\in C(G)^+$ such that

37 $$\begin{aligned}&\text{ Every } L_x f \quad (x\in G) \text{ lies } \text{ in } \text{ the } \text{ principal } \text{ ideal } C(G)_w; \nonumber \\&x\mapsto L_x f \text{ is } \text{ continuous } \text{ relative } \text{ to } \Vert \cdot \Vert _w. \end{aligned}$$

### Proof

Choose compact $K_1 \subset K_2 \subset \cdots$ such that $K_1$ is a neighbourhood of *e*; $K_n = K_n^{-1}$; $K_n K_n \subset K_{n+1}$, $\bigcup _{n\in \mathbb {N}} K_n = G$.

For $x\in G$, define [*x*] to be the smallest *n* with $x\in K_n$. Then $[x]=[x^{-1}]$, $[xy]\le 1 + [x]\vee [y]$ for all $x,y\in G$ by definition.

Let $f\in C(G)$. Define $u, v: G \rightarrow [1,\infty )$ as follows:

38 $$\begin{aligned} u(x) : = 1 + \sup |f(K_{n+1})|,\quad v(x) = [x] u(x) \quad \text{ if } [x]=n. \end{aligned}$$

1. If $x,y\in G$, $[x]\le [y]=n$, then $x,y\in K_n$, so
  39 $$\begin{aligned} |f(xy) | \le \sup |f(K_{n+1})|\le u(y). \end{aligned}$$
2. Hence, for all $x,y\in G$: $|f(x^{-1}y)|\le u(x) \vee u(y) \le u(x) v(y)$, i.e., $|L_x f| \le u(x) v$. Let $a\in G$, $\varepsilon >0$. In (5), using (3) and (4), we show the existence of a neighbourhood *U* of *e* with
  40 $$\begin{aligned} x\in a U \Longrightarrow | L_x f - L_a f | \le \varepsilon v. \end{aligned}$$
  Choose a *p* with $$a \in K_p$$.
3. $K_1$ contains an open set *V* containing *e*. We make a $q\in \mathbb {N}$ with
  41 $$\begin{aligned} x\in a V \Longrightarrow | L_x f - L_a f | \le \varepsilon v \text{ on } G{\setminus }K_q. \end{aligned}$$
  Let $x\in aV$. Then $x,a \in K_{p+1}$, so $[x],[a]\le p+1$. By (1):
  42 $$\begin{aligned} | (L_x f - L_a f)(y) | \le |f(x^{-1}y)| + |f(a^{-1}y)| \le 2 u (y) \text{ if } [y] \ge p+1. \end{aligned}$$
  Moreover, $\varepsilon v(y) = \varepsilon [y] u(y) \ge 2u (y)$ if $[y] \ge \frac{2}{\varepsilon }$. Take $q\in \mathbb {N}$ with $q\ge p+1$ and $q\ge \frac{2}{\varepsilon }$. For $y\in G {\setminus } K_q$ we have $[y]>q$, so $|(L_x f - L_a f)(y)| \le \varepsilon v(y)$.
4. We show there exists an open set *W* containing *e* with
  43 $$\begin{aligned} x\in aW \Longrightarrow | L_x f - L_a f | \le \varepsilon v \text{ on } K_q. \end{aligned}$$
  The function $(x,y) \mapsto | (L_x f - L_a f)(y) |$ on $G\times G$ is continuous and $K_q$ is compact. Hence by [16, Theorem 8.15] the function $G\mapsto [0,\infty )$
  44 $$\begin{aligned} x\mapsto \sup _{y\in K_q} | (L_x f - L_a f)(y) | \end{aligned}$$
  is continuous. Its value at *a* is 0, so there exists an open set *W* containing *e* with
  45 $$\begin{aligned} \sup _{y\in K_q} | (L_x f - L_a f)(y)| \le \varepsilon \quad (x\in a W). \end{aligned}$$
  As $v(y) \ge 1$ for all *y* we obtain (43).
5. With $U = V\cap W$ we have
  46 $$\begin{aligned} x\in aU \ \Longrightarrow \ |L_x f - L_a f|\le \varepsilon v \text{ on } G. \end{aligned}$$
  Therefore, to prove the theorem it is sufficient to show there exists a continuous function $w \ge v$:
6. Set $\alpha _n = n ( 1+ \sup | f(K_{n+1})| )$ for all *n*; then
  47 $$\begin{aligned}{}[x]=n \Longrightarrow v(x)= \alpha _n. \end{aligned}$$
  Put $K_0 = \emptyset$. For all $n\in \mathbb {N}$ we have $V K_{n-1} \subset K_1 K_{n-1} \subset K_n$, so $K_{n-1}$ is a subset of $K_n^\circ$, the interior of $K_n$. By Urysohn [8, Theorem VII.4.1 and Theorem XI.1.2] for all *n* there is a continuous $g_n : G \rightarrow [0,1]$ with
  48 $$\begin{aligned} g_n = 0 \text{ on } K_{n-1}, \ g_n = 1 \text{ on } G{\setminus } K_n^\circ \supset G {\setminus } K_n. \end{aligned}$$
  Let $b\in G$. There is a *l* with $b\in K_l$. *bV* is an open set containing *b*. As $bV \subset K_{l+1}$ and $g_n=0$ on $K_{n-1}$ we have: $g_n=0$ on *bV* as soon as $n\ge l+2$. Hence, $w:= \sum _{n\in \mathbb {N}}\alpha _{n+1} g_n$ is a continuous function $G \rightarrow [0,\infty )$. For every $x\in G$ there is an *n* with $[x]=n$; then $x\notin K_{n-1}$, $g_{n-1}(x)=1$ and $w(x) \ge \alpha _n = v(x)$. $\square$

### Theorem 9.2

Let *G* be a $\sigma$-compact locally compact group. Let $\mu$ be a finite measure on the Borel $\sigma$-algebra of *G* with a compact support. Let $\mathbb {B}$ be the Banach cover of *C*(*G*) consisting of the principal ideals as in Example 5.5. Then for every $f\in C(G)$ the function $x\mapsto L_x f$ is $\mathbb {B}$-integrable and its integral is the “convolution product” $\mu * f$:

49 $$\begin{aligned} (\mu * f) (y) = \int f(x^{-1}y) {{\mathrm{{\mathrm {d}}}}}\mu (x) \quad (y\in G). \end{aligned}$$

### Proof

By Theorem 9.1 there exists a $w\in C(G)^+$ such that (37) holds. This implies that the map $x \mapsto L_x f$ is Borel measurable and $\{L_x f : x\in G\}$ is separable in $C(G)_w$. As $x\mapsto \Vert L_x f\Vert _w$ is continuous and thus bounded on the support of $\mu$, the map $x \mapsto L_x f$ is $\mathbb {B}$-integrable (see 4.4(a)). That the integral is equal to $\mu * f$ follows by 4.4(c). $\square$

### Remark 9.3

Theorem 9.2 compares to [16, Example 8.17] in the sense that in both situations the convolution is equal to an integral of the translation. Though the situation is slightly different in the sense that in Theorem 9.2 we consider $\sigma$-compact locally compact groups, while in [16, Example 8.17] we considered metric locally compact groups (the fact that in [16, Example 8.17] $L_x f (t) = f(t x^{-1})$ is reminiscent for its statement).

## A Appendix: Absolutely dominating norms

**In this section,** $\varvec{C>0}$ **and** $\varvec{D}$ **is an ordered Banach space with closed generating cone and with a** $\varvec{C}$ **-absolutely dominating norm** $\varvec{\Vert \cdot \Vert }$ **.**

As is mentioned in 3.5, we show that $C\ge 1$ (A.1) and that for every $\varepsilon >0$ there exists an equivalent $(1+\varepsilon )$-absolutely norm (Theorem A.3). Furthermore, we discuss (in A.4–A.10) whenever there exists an equivalent norm $\Vert \cdot \Vert _1$ for which

50 $$\begin{aligned} \Vert x\Vert _1 = \inf \{ \Vert a\Vert _1 : a\in D^+, - a\le x \le a\} \quad (x\in D). \end{aligned}$$

This is done by means of the norm $\mathcal {N}$ introduced in A.2. Example A.11 illustrates that the existence of such equivalent norm may fail.

### A.1

Suppose that $C<1$. Choose $C'>0$ such that $C<C' <1$. For all $a\in D^+$ with $a\ne 0$ there exists a $b\in D^+$ with $a\le b$ and $\Vert b\Vert \le C' \Vert a\Vert$. Let $a\in D^+$ with $\Vert a\Vert =1$. Iteratively one obtains a sequence $a\le a_1 \le a_2 \le \cdots$ with $\Vert a_1\Vert \le C'$ and $\Vert a_{n+1}\Vert \le C' \Vert a_n\Vert$ for all *n*. Then $\sum _{n\in \mathbb {N}} \Vert a_n\Vert <\infty$ and thus $a_n \xrightarrow {u} 0$ by Theorem 3.9, which contradicts $0<a \le a_n$.

### A.2

Define $$\mathcal {N}: D \rightarrow [0,\infty )$$ by

51 $$\begin{aligned} \mathcal {N}(x) = \inf \{ \Vert a\Vert : a\in D^+, - a\le x \le a\}. \end{aligned}$$

$\mathcal {N}$ is a seminorm, and actually a norm because (see Theorem 3.9)

52 $$\begin{aligned} \mathcal {N}(x) = 0 \iff \text{ there } \text{ is } \text{ an } a^* \in D^+ \text{ with } - \tfrac{1}{n} a^* \le x \le \tfrac{1}{n} a^* \quad (n\in \mathbb {N}). \end{aligned}$$

Because $\Vert \cdot \Vert$ is *C*-absolutely dominating one has $\mathcal {N}\le C \Vert \cdot \Vert$.

### Theorem A.3

For all $\varepsilon >0$ there exists an equivalent norm $\rho$ on *D*, for which

53 $$\begin{aligned} (1+\varepsilon )^2 \rho (x) \ge \inf \{ \rho (a) : a\in D^+, - a \le x \le a\} \quad (x\in D). \end{aligned}$$

### Proof

Define $\rho := \varepsilon \mathcal {N}+ \Vert \cdot \Vert$. $\rho$ is a norm which is equivalent to $\Vert \cdot \Vert$, since $\mathcal {N}\le C\Vert \cdot \Vert$.

Let $x\in D$, $x\ne 0$. Choose $a\in D^+$ with $-a \le x\le a$ such that $\Vert a\Vert \le (1+\varepsilon ) \mathcal {N}(x)$. Note that by definition of $\mathcal {N}$ we have $\mathcal {N}(a)\le \Vert a\Vert$. Whence

54 $$\begin{aligned} \frac{\rho (a)}{\rho (x)} = \frac{ \mathcal {N}(a) + \varepsilon \Vert a\Vert }{ \mathcal {N}(x) + \varepsilon \Vert x\Vert } \le \frac{ \mathcal {N}(a) + \varepsilon \Vert a\Vert }{ \mathcal {N}(x) } \le \frac{ \Vert a\Vert + \varepsilon \Vert a\Vert }{ \mathcal {N}(x) } \le (1+\varepsilon ) \frac{\Vert a\Vert }{\mathcal {N}(x)} \le (1+\varepsilon )^2.\nonumber \\ \end{aligned}$$

### A.4

Suppose $\Vert \cdot \Vert _1$ is a norm equivalent to $\Vert \cdot \Vert$ for which there exists a $C>0$ such that

55 $$\begin{aligned} C\Vert x\Vert _1 = \inf \{ \Vert a\Vert _1 : a\in D^+, - a\le x \le a\} \quad (x\in D). \end{aligned}$$

Then it is straightforward to show that $\mathcal {N}$ is equivalent to $\Vert \cdot \Vert$.

### A.5

$\mathcal {N}$ is equivalent to $\Vert \cdot \Vert$ if and only if there exists a $c>0$ such that $\mathcal {N}\ge c\Vert \cdot \Vert$. The latter is true if and only if

56 $$\begin{aligned} -\tfrac{1}{n} a^* \le x_n \le \tfrac{1}{n} a^* \quad (n\in \mathbb {N}) \quad \Longrightarrow \quad \lim _{n\rightarrow \infty }\Vert x_n\Vert =0. \end{aligned}$$

### Theorem A.6

57 $$\begin{aligned} \mathcal {N}(x) = \inf \{ \mathcal {N}(a): a\in D^+, -a \le x \le a\}. \end{aligned}$$

### Proof

($\le$) Take $a\in D^+, -a \le x \le a$; we prove $\mathcal {N}(x) \le \mathcal {N}(a)$. For all $b\ge a, -b \le x \le b$, whence $\mathcal {N}(x) \le \Vert b\Vert$. Thus $\mathcal {N}(x) \le \inf \{\Vert b\Vert : a\le b\} = \mathcal {N}(a)$, the latter by definition of $\mathcal {N}(a)$.

($\ge$) Take $\varepsilon >0$. Choose $a\in D^+, -a \le x\le a$, $\Vert a\Vert \le \mathcal {N}(x) + \varepsilon$. Then $\mathcal {N}(a) \le \Vert a\Vert \le \mathcal {N}(x) +\varepsilon$.

### Theorem A.7

[1, Theorem 2.38] For an ordered normed vector space *E* the following are equivalent.

- The cone $E^+$ is normal.
- The normed space *E* admits an equivalent monotone norm.
- There is a $c>0$ such that $0\le x\le y$ implies $\Vert x\Vert \le c\Vert y\Vert$.

### A.8

Suppose *D* is an ordered Banach space with closed generating cone and suppose there exists a $c>0$ such that

58 $$\begin{aligned} \Vert x\Vert \le c \inf \{ \Vert a\Vert : a\in D^+, -a \le x \le a\} = c \mathcal {N}(x). \end{aligned}$$

Then for $x\in D^+$, $a\in D^+$ with $x\le a$ one has $\Vert x\Vert \le c\Vert a\Vert$.

### A.9

Suppose *D* is an ordered Banach space with closed generating cone and $\Vert \cdot \Vert$ is monotone. Let $x\in D$ and $a\in D^+$ be such that

59 $$\begin{aligned} -a \le x \le a. \end{aligned}$$

Then $0 \le x+a \le 2a$ and whence $\Vert x\Vert \le \Vert x+a\Vert + \Vert a\Vert \le 3\Vert a\Vert .$ Thus

60 $$\begin{aligned} \Vert x\Vert \le 3 \inf \{ \Vert a\Vert : a\in D^+, -a \le x \le a\} = 3 \mathcal {N}(x). \end{aligned}$$

We conclude:

### Theorem A.10

Let *D* be an ordered Banach space with closed generating cone. The following are equivalent

- There exist a norm $\Vert \cdot \Vert _1$ that is equivalent to $\Vert \cdot \Vert$ for which
  61 $$\begin{aligned} \Vert x\Vert _1 = \inf \{ \Vert a\Vert _1 : a\in D^+, - a\le x \le a\} \quad (x\in D). \end{aligned}$$
- There exists a $c>0$ such that $\mathcal {N}\ge c\Vert \cdot \Vert$.
- There exists a monotone norm that is equivalent to $\Vert \cdot \Vert$.
- $$E^+$$ is normal.

In the following we give an example of an ordered Banach space with closed generating cone *D* for which none of (a)–(d) of Theorem A.10 holds.

### Example A.11

Let *D* be $\ell ^1$ with its natural norm. Define $T: \ell ^1 \rightarrow \mathbb {R}^\mathbb {N}$ by

62 $$\begin{aligned} Tx = (x_1,x_1+ x_2, x_1+ x_2+ x_3,\ldots ). \end{aligned}$$

As *T* is linear, *D* is an ordered vector space under the relation $\preceq$

63 $$\begin{aligned} x \preceq y \iff Tx \le Ty. \end{aligned}$$

The positive cone of $\ell ^1$ is included in $D^+$, whence *D* is directed. Moreover, $D^+$ is closed and so $\Vert \cdot \Vert$ is absolutely dominating. With $x_n = (1,-1,1,-1,\ldots , \pm 1, 0,0 \ldots )$ and $a=(1,0,0,\ldots )$ we have $- a \le x_n \le a$ and $\Vert x_n\Vert =n$, $\Vert a\Vert =1$.

## B Appendix: Banach cover of Köthe spaces

**In this section** $\varvec{(Y,\mathcal {B},\nu )}$ **is a complete** $\varvec{\sigma }$ **-finite measure space and** $\varvec{M}$ **is the space of classes of measurable functions** $\varvec{Y \rightarrow \mathbb {R}}$ **as in Example** 5.6.

### Lemma B.1

$\{L_{\rho _w} : w\in M^+, w>0\}$ a.e. is a Banach cover of *M*.

### Proof

$L_{\rho _w}$ is complete since $f\mapsto fw$ is an isometric bijection $M_w \rightarrow L^1(\nu )$.

Let $$f\in M$$. We show there exists a $$w\in M^+$$, $$w>0$$ a.e., with $$f\in L_{\rho _w}$$. By the $$\sigma $$-finiteness of $$\nu $$ there is a $$u\in L^1(\nu )$$, $$u>0$$ a.e. Put $$w = (|f|+1)^{-1}u $$. Then $$w\in M^+$$, $$w>0$$ a.e., and $$f\in L_{\rho _w}$$ because $$\int |f| w {{\mathrm{{\mathrm {d}}}}}\nu \le \int u {{\mathrm{{\mathrm {d}}}}}\nu <\infty $$.

If $w, v\in M^+$, $w>0, v>0$ a.e., then $w\wedge v>0$ a.e., $L_{\rho _{w\wedge v}}$ is a subset of $L_{\rho _w}$ and $L_{\rho _v}$ and $\rho _{w\wedge v} \le \rho _{w}$ on $L_{\rho _w}$ and $\rho _{w \wedge v} \le \rho _v$ on $L_{\rho _v}$. $\square$

### Theorem B.2

The complete Köthe spaces form a Banach cover of *M*.

### Proof

Let $\rho$ be a function norm and $L_\rho$ be complete. If $f_n \in M^+$ for $n\in \mathbb {N}$ and $\sum _{n\in \mathbb {N}}\rho (f_n) <\infty$, then $\sum _{n\in \mathbb {N}}|f_n| \in L_\rho$, so $\sum _{n\in \mathbb {N}}|f_n|<\infty$ a.e. and $f_n \rightarrow 0$ a.e. We will use this fact below.

By Lemma B.1 it suffices to prove the following. Let $\rho _1, \rho _2$ be function norms, $L_{\rho _1}$ and $L_{\rho _2}$ complete. We make a function norm $\rho$ such that $L_\rho$ is complete and $\rho \le \rho _1$, $\rho \le \rho _2$. (Then $L_{\rho _1}, L_{\rho _2} \subset L_\rho$ and we are done.)

Define $$\rho : M\rightarrow [0,\infty ]$$ by

64 $$\begin{aligned} \rho (f) = \inf \{ \rho _1(g) + \rho _2(h) : g,h\in M^+, g+h \ge |f| \}. \end{aligned}$$

If $\rho (f) =0$, choose $g_n, h_n$ with $g_n + h_n \ge |f|$, $\rho _1(g_n) + \rho _2(h_n) \le 2^{-n}$. Then (by the above), $g_n \rightarrow 0$ a.e., $h_n \rightarrow 0$ a.e. Hence, $f=0$ a.e. It follows easily that $\rho$ is a function norm. Obviously, $\rho \le \rho _1$, $\rho \le \rho _2$. For the completeness of $L_\rho$: Let $u_1,u_2,\ldots \in L_\rho ^+$, $\sum _{n\in \mathbb {N}}\rho (u_n) <\infty$. Choose $g_n, h_n \in M^+$, $g_n + h_n \ge u_n$, $\rho _1(g_n) + \rho _2(h_n) < \rho (u_n) + 2^{-n}$. Then $\sum _{n\in \mathbb {N}}\rho _1(g_n) <\infty$, so $\sum _{n\in \mathbb {N}}g_n \in L_{\rho _1}$, $\rho _1( \sum _{n\in \mathbb {N}}g_n) <\infty$. Similarly $\rho _2(\sum _{n\in \mathbb {N}}h_n) <\infty$. Then $\sum _{n\in \mathbb {N}}u_n \in L_\rho$.

## Footnotes

1
$D^+$ is generating if $D=D^+ - D^+$.

2
*D* is directed if $D=D^+- D^+$, i.e., if $D^+$ is generating.

3 Batty and Robinson [3] call the cone $D^+$ *approximately C-absolutely dominating*, and, Messerschmidt [13] calls *D* *approximately C-absolutely conormal*, if the norm on *D* is *C*-absolutely dominating.

4 For normed Riesz spaces property (ii) is equivalent to what is called the weak Riesz-Fischer condition (see Zaanen [19, Ch. 14, §101]).

5 For complete metrisable ordered vector spaces.

6 Even though we use the same notation as in Sect. 4, see 4.3, the meaning of ${\mathfrak {B}}_D$ is slightly different.

7 We write $E^\sim$ for the space of order bounded linear maps $E\rightarrow \mathbb {R}$.

8 In this section we close an eye for the difference between a function and its equivalence class. There will be no danger of confusion.
